# In vivo molecular imaging in preclinical research

**DOI:** 10.1186/s42826-022-00142-3

**Published:** 2022-10-21

**Authors:** Su Jin Kim, Ho-Young Lee

**Affiliations:** 1 Department of Nuclear Medicine, Seoul National University Bundang Hospital, Seongnam, South Korea; 2 Department of Nuclear Medicine, Seoul National University College of Medicine, Seoul, South Korea

**Keywords:** In vivo Molecular Imaging, Fluorescence, Bioluminescence, Computed tomography, PET/CT, SPECT/CT, Probe, Mouse, Small animal imaging

## Abstract

In vivo molecular imaging is a research field in which molecular biology and advanced imaging techniques are combined for imaging molecular-level biochemical and physiological changes that occur in a living body. For biomolecular imaging, the knowledge of molecular biology, cell biology, biochemistry, and physiology must be applied. Imaging techniques such as fluorescence, luminescence, single-photon emission computed tomography (SPECT), positron emission tomography (PET), computed tomography (CT), and magnetic resonance imaging (MRI) are used for biomolecular imaging. These imaging techniques are used in various fields, i.e., diagnosis of various diseases, development of new drugs, development of treatments, and evaluation of effects. Moreover, as biomolecular imaging can repeatedly acquire images without damaging biological tissues or sacrificing the integrity of objects, changes over time can be evaluated.

Phenotypes or diseases in a living body are caused by the accumulation of various biological phenomena. Genetic differences cause biochemical and physiological differences, which accumulate and cause anatomical or structural changes. Biomolecular imaging techniques are suitable for each step. In evaluating anatomical or structural changes, MRI, CT, and ultrasound have advantages in obtaining high-resolution images. SPECT and MRI are advantageous for the evaluation of various physiological phenomena. PET and magnetic resonance spectroscopy can be used to image biochemical phenomena in vivo. Although various biomolecular imaging techniques can be used to evaluate various biological phenomena, it is important to use imaging techniques suitable for each purpose.

## Background

In vivo molecular imaging is an advanced imaging approach that combines biochemical and physiological changes occurring in living animals at the molecular level [1, 2]. Fluorescence [3, 4], luminescence [5, 6], single-photon emission computed tomography (SPECT) [7], positron emission tomography (PET) [8, 9], computed tomography (CT) [10, 11], and magnetic resonance imaging (MRI) are techniques used to obtain images [12, 13]. Each imaging method has advantages and disadvantages.

**Fig. 1:**
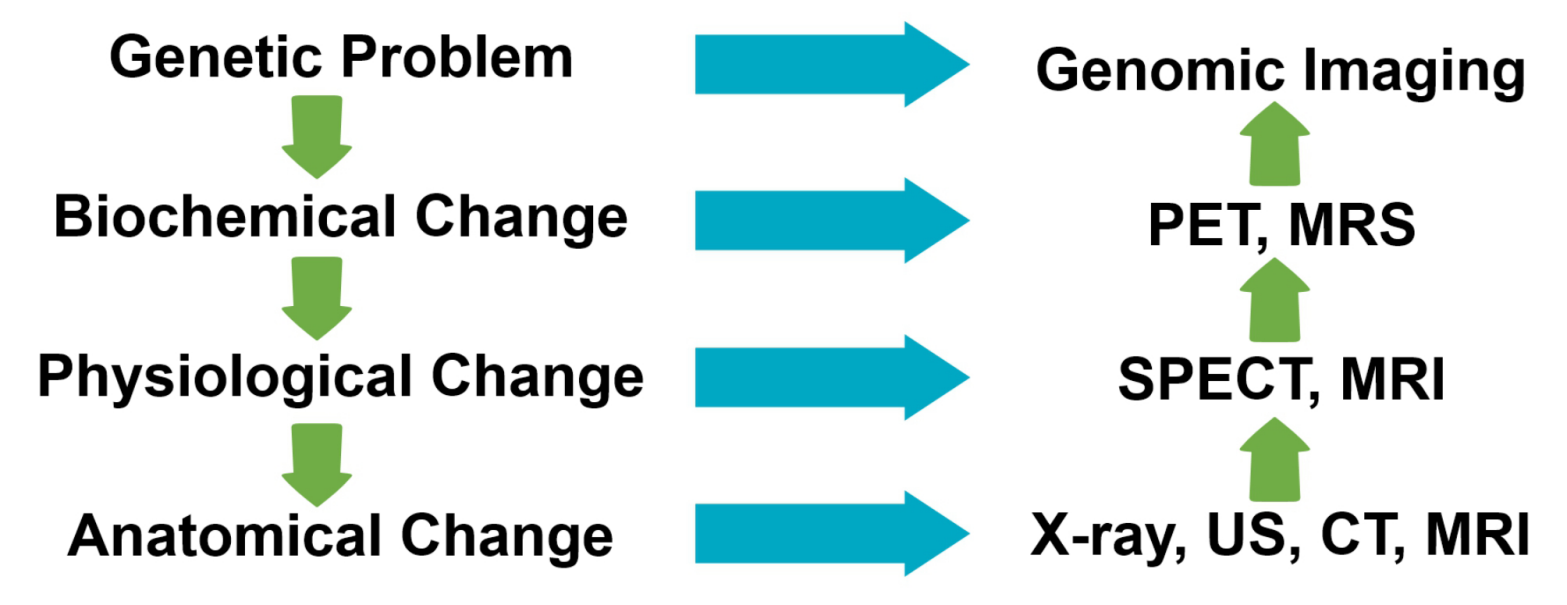
Molecular imaging technology for biological phenomena

A disease or phenotype in a living organism is the result of different biological events. Genetic variations in the body can lead to biochemical or physiological variations. Accumulation of these variations results in structural or anatomical alterations. Scientists should employ the most suitable biomolecular imaging methods for their research themes (Fig. 1).

Genetic alteration is the first step toward a disease or change in a living organism. Biochemical and physiological alterations result from accumulation of these genetic mutations. Anatomical alterations result from accumulation of these changes. To research the occurrence or diagnosis of a disease, appropriate imaging techniques each stage must be used or developed.

High-resolution images can be acquired using CT and MRI. Thus, it is appropriate to assess anatomical structures. CT and MRI have low sensitivity for evaluating biochemical processes with a high resolution. The sensitivity in this article means the detection efficiency of the biological process. The sensitivity is qualitative term referring to the size of the smallest detail that can be recorded and discernible on imaging and widely used in radiology. Optical bioimaging techniques, such as fluorescence and bioluminescence imaging, are extremely sensitive with high signal-to-noise ratios for the evaluation of biological processes. Despite excellent sensitivity, its quantification and tissue depth are limited. Optical imaging is unable to assess cellular activity in the deep region of a living body. Radiopharmaceuticals are used in nuclear medicine imaging to assess the metabolic or physiological processes occurring within a living body. Nuclear medicine imaging methods such as SPECT/CT and PET/CT can be employed in preclinical studies. SEPCT/CT or PET/CT offers a lower anatomical resolution and higher functional sensitivity than CT and MRI. These methods are less sensitive than optical imaging; however, they have great quantification and no tissue depth restrictions. Using a probe or radiopharmaceutical gives new meaning to optical imaging or nuclear medicine imaging. In this article, we introduce various in vivo molecular imaging techniques to understand each of their advantages and disadvantages (Table 1). The aim of this article is to help researchers use suitable in vivo molecular imaging techniques for the acceleration of numerous studies.

**Table 1:** Characteristics of molecular imaging modalities

Imaging techniques	Resolution	Depth information	Acquisition time	Sensitivity
CT	50 μm	No limit	min	Not characterized
MRI	10–100 μm	No limit	min–h	10^− 3^ ~ 10^-5^ mol/L
Bioluminescence	1–2 mm	< 1–2 cm	min	10^–15^ ~ 10^-17^ mol/L
Fluorescence	1–2 mm	< 1 cm	s– min	10^− 9^ ~ 10^-12^ mol/L
SPECT	0.5–2 mm	No limit	min	10^–10^ ~ 10^-11^ mol/L
PET	0.1–1 mm	No limit	min	10^–11^ ~ 10^-12^ mol/L

## Main text

### Bio-optical imaging techniques

Microscopy used for in vitro or ex vivo purposes is the most popular optical imaging technology in the field of biological imaging. The primary application of microscopy is in the investigation of cellular phenomena. We can image useful biological information in the living body in real time by using this optical approach.

As bioluminescence probes, fluorescence probes, diverse functional fluorescence probes, and fluorescence nanoparticles are being created to target particular biological phenomena, optical molecular imaging is developing at a rapid rate. Optical imaging has the benefit of having a reasonably quick capture time and good sensitivity compared to other biomolecular imaging techniques.

The most crucial technical aspect of optical bioimaging is the ability to accurately detect light generated by a living organism. A charge-coupled device (CCD) camera is available for detecting low light with extremely high sensitivity. The surface of a silicon chip is divided into numerous light-sensitive pixels in CCD cameras. Owing to the extremely high sensitivity of CCD cameras, they are transformed into photons with wavelengths of 300–1,000 nm and energies of 2–3 eV, which are subsequently amplified to capture images. The silicon lattice generated heat during this process. Heat noise, in which electrons are continuously released as a result of thermal energy, is a crucial factor impeding the sensitivity of the CCD. Previously, optical imaging equipment used a stationary CCD camera that was attached to a refrigerator to keep it cool, situated in a darkroom. Recently, equipment and darkrooms for animal photography have been combined, and the camera now includes the cooling system of the CCD camera. What matters is how low the concentration must be to prevent the CCD camera from overheating. These CCD cameras have recently been improved; however, they still have several substantial drawbacks.

First, the animals were opaque, which resulted in a low light transmission efficiency. The transmission efficiency varies with the type of tissue, and the image is considerably affected by the light scattering that occurs in different types of tissue. The presence or absence and type of mouse fur have a major impact on the optical image. Second, there is no information regarding the depth of the image signal because the image created by the CCD camera is essentially two-dimensional. A rotating CCD camera, or a technique for capturing images from various angles with a single CCD camera, is being developed or used to solve these problems [14]. These limitations should be considered when designing experiments that use bio-optical imaging.

## In vivo bioluminescence imaging

**Fig. 2:**
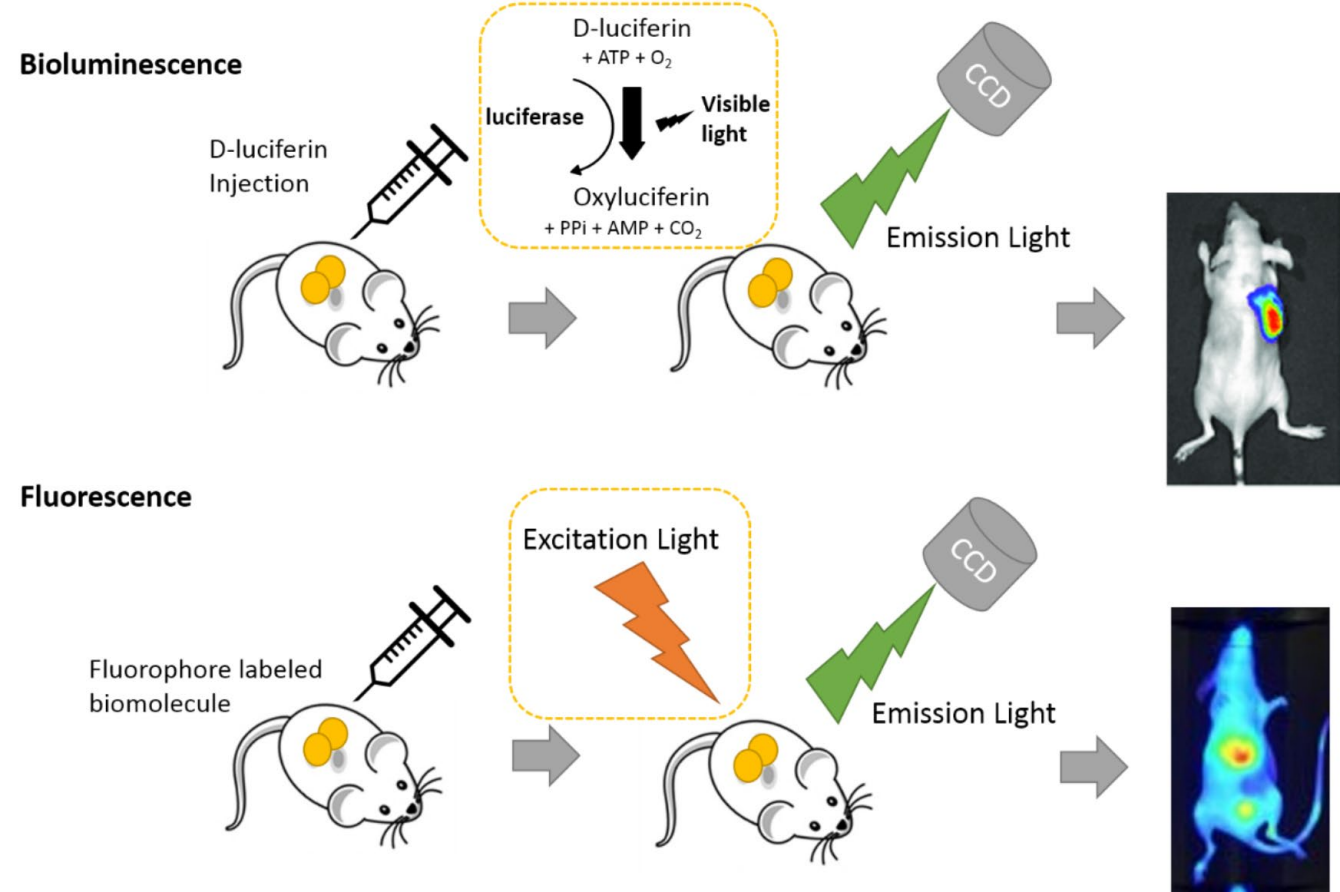
Basic principles of fluorescence and bioluminescence

A biological entity that will be analyzed by bioluminescence optical imaging must be genetically modified to express a light-producing enzyme (luciferase). Crustaceans, fish, bacteria, and other species also contain luciferase enzymes. This enzyme produces light by oxidizing the enzyme-specific substrate with oxygen and ATP. This reaction generates chemical energy, some of which is released as visible light (Fig. 2). This chemiluminescence reaction occurs only in living cells, in which luciferase is expressed by genetic manipulation. Therefore, the greatest advantage of bioluminescence optical imaging is the absence of a background signal in the image, and the image signal is only generated in cells in which the matrix-enzyme reaction occurs. Therefore, the specificity and sensitivity for detecting even very low signals are much higher than other imaging techniques.

Light produced by luciferase in vivo is absorbed and scattered in tissue before leaving the body. Hemoglobin is the primary component that absorbs light in the body. It is primarily present in red blood cells and absorbs light at a wavelength < 600 nm in the blue and green spectra. In vitro luciferase light is primarily present in the red spectrum between 600 and 1,000 nm. *Photinus pyralis* firefly luciferase (Fluc), a variety of luciferases, can generate light with a diverse spectrum of wavelengths. Light at a wavelength of 560 nm and a considerable amount of light in the spectrum above 600 nm were included, making it suitable for bioluminescence optical imaging. Fluc reacts with D-luciferin, a specific substrate, oxidizes it to oxyluciferin, and emits light. This sequence of processes is possible in the presence of oxygen, magnesium, and adenosine triphosphate (ATP). Bioluminescence optical imaging has several characteristics for using enzyme-substrate reactions. First, there is no need to irradiate light from the outside to activate luciferase. It generates light in the presence of substrates D-luciferin, ATP, oxygen, and magnesium. Second, if the substrate D-luciferin is present, the circulating speed of the enzyme is increased and the enzyme does not accumulate in the cell; therefore, it is possible to repeatedly acquire images every few hours. Finally, there is a positive correlation between the concentration of the enzyme and the intensity of the light generated. While these characteristics exist, there are also points to be aware of for use in experiments. To use the bioluminescence optical acquisition method in an experiment, it is necessary to understand the biological phenomenon to be evaluated and to decide what to use as a reporter gene for this series of biological processes. It is important to acquire images at various intervals following injection of D-luciferin, in the initial stage, to determine the best image time point when comparing images of the same subject over several hours or acquiring images once. The time required for injected D-luciferin to reach the target cells is extremely important. If an image is acquired without understanding its kinetics, there is a high possibility that an appropriate image cannot be obtained.

Firefly luciferase and luciferin were used for the bioluminescence imaging. Therefore, it is necessary to use genetically modified organisms or cells to express luciferase in the mechanism under study. Image evaluation is also affected by the intraperitoneal injection of luciferin to reach the region of interest. A fluorescent substance or protein is used in biofluorescence imaging. The light produced by the fluorescent material excited by the outside light was used to acquire the image. The experiment should be planned considering the autofluorescence that can arise depending on the tissue, owing to external light.

## In vivo fluorescence imaging

A CCD camera is used for fluorescence imaging, which involves exposing fluorescent proteins and nanoparticles to light of a specific wavelength that excites them. The excited fluorescent proteins, nanoparticles, etc., subsequently emit light of a specific wavelength, which is captured and imaged by the CCD camera. Biofluorescent optical imaging can be performed in several ways. There is a technique for capturing pictures of an animal or cell that expresses a fluorescent protein gene [15]. Another technique involves injecting a probe with a fluorescent protein, a fluorescent material, or nanoparticles to obtain a picture [16]. The test strategy should be selected based on the goals of the experiment. Green light with a wavelength of 509 nm is produced by green fluorescence protein (GFP), which is stimulated by light of a wavelength of 395 nm. However, GFP has limitations when collecting bioimages overlaid with mouse skin autofluorescence. Proteins or fluorophores that produce longer-wavelength fluorescence have been created and employed to overcome these restrictions. In particular, when imaging deep tissues or cells, the transmission of excitation light should be considered when designing biofluorescence optical imaging investigations [17]. When applying the biofluorescence optical imaging technique to a mouse, the fur of the mouse should be considered. However, it is difficult to apply biofluorescence optical imaging to hairy animals. A good method is to remove the hair from a specific area of interest. Alternatively, it should be checked that the color of the fur does not interfere with the excitation light or the light for excitation. In addition, the transmittance of light should be considered when a deep region of interest is located. In example, the signal of GFP protein could overlap with the autofluorescence of the skin. To avoid the overlapping the signals, the long wavelength emission fluorophore should be used. In case to evaluate the biological process in the deep tissue, the penetrating ability of exiting light and emission light should be considered. In that cases, the near infrared fluorophore should be considered.

## Biomolecular imaging using single-photon emission computed tomography

Planar pictures and tomography are the two main categories of image-acquisition techniques that employ gamma rays. Depending on how gamma rays are detected, tomography can be divided into single-photon tomography and PET. Gamma rays are detected using single-photon tomography rather than PET.

Pin-hole collimators can be used with gamma images to solve the resolution problem of planar images. However, a pinhole collimator has the disadvantage of lengthy acquisition time, which prevents it from producing high-resolution images. Furthermore, when utilizing a pinhole collimator, the sensitivity decreases, and the spatial resolution improves as the distance increases, decreasing the effectiveness of the image acquisition system. The sensitivity improves as the pinhole increases; however, the spatial resolution increases. Low spatial resolution and high sensitivity are required for the imaging of small animals. A multi-pinhole collimator-based SPECT/CT scanner for small animals was created and used to address these disadvantages [18]. The resolution of SPECT is increased to < 1 mm, and the sensitivity is increased by utilizing a multi-pin hole collimator, allowing the expansion of the effective field of view.

## Biomolecular imaging using positron emission tomography

PET is a radiopharmaceutical bound to a radioactive isotope that emits positrons injected into the body. The injected radiopharmaceutical is traced using a PET machine to track its distribution, biology, and biochemistry. It is a method for imaging and quantitative analysis of biochemical or functional characteristics. The positron emitted from the radiopharmaceutical meets the surrounding electrons and annihilation occurs. At this time, as the mass of the two electrons disappears, a corresponding 511 keV is generated in both directions by 180 °. A PET image can be obtained by reconstructing an image of the generated gamma rays using gamma rays detected simultaneously by a circular detector of a PET scanner [19].

In PET, the biochemical or physiological significance of the image differs depending on the radiopharmaceutical used (Table 2). Medical cyclotrons are required to produce radiopharmaceuticals for PET. This is necessary to produce a positron-emitting radioisotope, and a hot cell, a synthesis device, etc., are required to synthesize various radiopharmaceuticals using the produced radioisotope.

**Table 2:** Summary of SPECT imaging tracer

Radioisotope	Energy (keV)	Half life	Radiopharmaceuticals
^99m^Tc	144	6.0 h	^99m^Tc-MDP (bone metastases)
			^99m^Tc-MIBI (myocardial perfusion)
			^99m^Tc-HMPAO (brain perfusion)
			^99m^Tc-ECD (brain perfusion)
			^99m^Tc-DMSA (renal cortex)
			^99m^Tc-DTPA (renal function)
			^99m^Tc-MAG3 (renal function)
			^99m^Tc-HSA (lymphatic fluid)
^125^I	35	59.6 d	^125^I (thyroid)
			^125^I-antibody
			^125^I-protein
^123^I	159	13.2 h	^123^I (thyroid)
			^123^I-MIBG (renal function)
			^123^I-IudR (cell proliferation)

## Imaging using surface-enhanced Raman scattering (SERS)

Raman scattering, often known as the Raman effect, is a type of scattering that modifies the wavelength of light. It was first described in 1928 by C.V. Raman, and Krishnan made the initial discovery. A portion of the light that is scattered when it passes through a certain medium preserves its original energy, although it occasionally has more or less energy than the initial light energy. Rayleigh scattering is the process of dispersing light while retaining its initial energy, whereas Raman scattering is the process of scattering light while losing or gaining energy. The Stokes band position and number of molecules can be used to calculate the energy of the vibration mode. Owing to the relatively low energy of Raman scattering, nanoparticles are necessary to enhance the Raman signal for in vivo imaging [20]. In addition, SERS has a relatively low bandwidth compared with fluorescence. Therefore, it can be used for characterizing multiple biosignal evaluations. SERS uses plasmonic nanoparticles with the Raman effect to generate an extremely intense and recognized spectrum that mimics a fingerprint [21]. SERS nanoparticles provide a platform with a range of opportunities, including multimodality and unparalleled sensitivity owing to surface-enhanced resonance Raman spectroscopy and molecular resonance effects, active molecular targeting via surface functionalization with targeting moieties, multiplexed imaging, where the spectra of different Raman reporter molecules serve to reveal a specific marker, and multimodality, where the metal core (typically gold) may simultaneously serve as a CT, MRI contrast agent or PET, and SPECT agents [22–24].

Using multimodal nanoparticles, the in vivo affinity of antibodies targeting the same epitope can be evaluated. In the process of developing an antibody for an epitope, there could be more than two candidate antibodies. Antibody affinity was evaluated using in vitro methods. However, the in vivo status was different from that in the in vitro experimental situation. Discrepancies between the two situations were frequently detected. By administrating nanoparticles labeled with different antibodies targeting the same epitope, the in vivo affinity of each antibody could be evaluated in exactly the same situation. Each antibody can be differentiated by the SERS signal from the nanoparticles [25].

## Conclusion

Small animals have been used in several experiments. In vitro experiments were used to evaluate the biological processes in the cell or tissue at one time point. Therefore, subsequent evaluation of the biological process requires many mice. However, using in vivo molecular imaging, we could investigate sequential biological phenomena, with the exception of individual variations. Although various biomolecular imaging methods can be used to evaluate various biological phenomena, as described above, each imaging method has advantages and disadvantages. Therefore, it is important to use imaging techniques that are suitable for each research purpose.

## Data Availability

Not applicable.

## Abbreviations

CCD: charged-coupled device
CT: computed tomography
Fluc: *Photinus pyralis* firefly luciferase
GFP: green fluorescent protein
MRI: magnetic resonance imaging
PET: positron emission tomography
SPECT: single-photon emission computed tomography
